# I trust my immunity more than your vaccines: “Appeal to nature” bias strongly predicts questionable health behaviors in the COVID-19 pandemic

**DOI:** 10.1371/journal.pone.0279122

**Published:** 2023-02-22

**Authors:** Iris Žeželj, Marija Petrović, Anja Ivanović, Predrag Kurčubić

**Affiliations:** 1 Faculty of Philosophy, Department of Psychology, University of Belgrade, Belgrade, Serbia; 2 Faculty of Philosophy, LIRA Laboratory for Individual Differences, University of Belgrade, Belgrade, Serbia; 3 Ipsos Strategic Marketing, Belgrade, Serbia; Xiamen University - Malaysia Campus: Xiamen University - Malaysia, MALAYSIA

## Abstract

Health care policies often rely on public cooperation, especially during a health crisis. However, a crisis is also a period of uncertainty and proliferation of health-related advice: while some people adhere to the official recommendations, others tend to avoid them and resort to non-evidence based, pseudoscientific practices. People prone to the latter are often the ones endorsing a set of epistemically suspect beliefs, with two being particularly relevant: conspiratorial pandemic-related beliefs, and the appeal to nature bias regarding COVID-19 (i.e., trusting natural immunity to fight the pandemic). These in turn are rooted in trust in different epistemic authorities, seen as mutually exclusive: trust in science and trust in the “wisdom of the common man”. Drawing from two nationally representative probability samples, we tested a model in which trust in science/wisdom of the common man predicted COVID-19 vaccination status (Study 1, N = 1001) or vaccination status alongside use of pseudoscientific health practices (Study 2, N = 1010), through COVID-19 conspiratorial beliefs and the appeal to nature bias regarding COVID-19. As expected, epistemically suspect beliefs were interrelated, related to vaccination status, and to both types of trust. Moreover, trust in science had both a direct and indirect effect on vaccination status through both types of epistemically suspect beliefs. Trust in the wisdom of the common man had only an indirect effect on vaccination status. Contrary to the way they are typically portrayed, the two types of trust were unrelated. These results were largely replicated in the second study, in which we added pseudoscientific practices as an outcome; trust in science and the wisdom of the common man contributed to their prediction only indirectly, through epistemically suspect beliefs. We offer recommendations on how to make use of different types of epistemic authorities and how to tackle unfounded beliefs in communication during a health crisis.

"But if you’re like 21 years old, and you say to me, ‘Should I get vaccinated?’ I’ll go, ‘No. Are you healthy? Are you a healthy person? Like, look, don’t do anything stupid, but you should take care of yourself. If you’re a healthy person, and you’re exercising all the time, and you’re young, and you’re eating well, I don’t think you need to worry about this."

            Joe Rogan at The Joe Rogan Experience, April 23, 2021

## Introduction

People make hundreds of decisions per day; many of them are directly or indirectly health-related [1]. Furthermore, often they not only decide for themselves, but their decisions impact other people’s health. Implementing public health policies typically requires individual cooperation [2]. Such cooperation, however, is rarely unanimous. There is always a portion of individuals who avoid following the recommended practices; the same people sometimes resort to non-evidence-based traditional, complementary and alternative (TM/CAM) health practices (see [3] for a review). In this paper, we investigate the relations between these two broad types of questionable health behaviors and their psychological roots.

The reason why not adhering to official medical recommendations is detrimental to public health is rather self-evident: it leads to increased mortality rates [4], spread of infectious diseases [5], and economic burden on healthcare systems [6]. The World Health Organization reports that prevalence of non-adherence for chronic diseases is as high as 50% [7], and vaccine hesitancy and antibiotic overuse were listed in top 10 global health risks even before the pandemic [8]. On the other hand, while often perceived as harmless, non-evidence-based TM/CAM practices can be just the opposite: acupuncture may cause infections, alternative diets can cause malnutrition, chiropractic spinal manipulations can cause a stroke, herbal remedies can cause liver damage [9]. The onset of the pandemic only saw an increase of such non-evidence-based pseudoscientific advice [10]. It became so pervasive, that WHO had to set up a “mythbusters” website page devoted to debunking different non-evidence-based practices for combating COVID-19 [11], from constantly sipping liquid, rinsing nose with saline solutions, traditional cures such as consuming garlic, honey or hot peppers, to drinking methanol, ethanol or bleach.

Furthermore, non-evidence-based TM/CAM practices can also be accompanied by delay or avoidance of official treatment: opting for a TM/CAM treatment is related to increased mortality of cancer patients [12]. They can also interact with official treatment, proven risky for cancer patients [13] and HIV patients [14]. There is anecdotal evidence that people prone to these practices are less likely to follow recommended ones when it comes to the COVID-19 pandemic as well: for example, Dr Nestorović, a renown Serbian pulmonologist and a vocal public figure famously revealed that he does not need the COVID-19 vaccine because he keeps his immunity strong with a quince jam and homemade brandy combination [15]; Joe Rogan, the host of one the most popular US podcasts “The Joe Rogan Experience”, was also vocal about recommending young people who “work on their immunity” by exercising regularly, eating healthy and taking supplements not to take the vaccine [16]. We argue that these health behaviors can be traced back to a set of so-called “epistemically suspect” beliefs [17], and we especially focus on two: pandemic-related conspiracy theories and the appeal to nature bias.

### Conspiracy theories and health behaviors

As conspiracy theorizing always involves questioning the official versions of events [18], it should also translate to questioning the intentions of the officials, including their health recommendations. There is ample evidence that believing in conspiracy theories is related to less adherence to recommended health practices, such as less frequent physical and dental checkups and use of sunscreen [19]. Exposure to anti-vaccine conspiracy theories has been shown to decrease vaccination intentions [20]. During the 2020–21 COVID-19 pandemic, pandemic-related conspiracy beliefs have repeatedly been linked to vaccine hesitancy (see [21] for a review).

Conspiracy mentality was also shown to be positively related to the appreciation of alternative medicine in general [22], and people more prone to believing in conspiracy theories endorsed CAM products more than those less prone to it [23,24]. There is also scattered evidence that those who endorse pandemic-related conspiracy beliefs are also more likely to believe that pseudoscientific cures, such as consuming megadoses of garlic or vitamins, sipping ginger potions or drinking colloidal silver can prevent or cure COVID-19 [25]. Not only that, but believing in conspiracy theories about the pandemic also leads to more frequent use of the same practices [26].

### Appeal to nature and health behaviors

David Hume famously argued that an ‘ought’ cannot be logically derived from an ‘is’, and ‘ought not’ derived from ‘is not’ [27]. Following this observation, Moore warned that there is no merit in explaining something as being good reductively, evoking its natural properties such as desirable or pleasant, i.e., that doing so would be committing a naturalistic fallacy [28]. It has since been used in a broader sense, as the idea that what is found in nature is necessarily good/beneficial (and what is not is bad/harmful), and sometimes labeled as the “appeal to nature” bias [29]. This bias was observed to be central for a number of anti-scientific attitudes and behaviors, such as rejecting GMO food [30] or opposing in vitro fertilization [31]. There are authors (e.g. [32]) claiming that this bias is even embedded in the official legislation, for example in the EU regulations on organic food products.

The appeal to nature bias is also very present in anti-vaccinal narratives, either through claims that vaccine-induced immunity is inferior to natural immunity, acquired post-disease, or through claims that vaccines are unnecessary if one maintains their immunity by healthy life habits [33,34]. Similar tropes were evoked on social media during the COVID-19 pandemic, with users claiming that natural immunity is “99.9% effective” or their immune system is “99.4% effective” against the virus, alluding to vaccine efficacy [35].

### Trust in epistemic authorities

Digging even deeper, these beliefs can be rooted in trust in different epistemic authorities–science, on the one hand, and the so-called “wisdom of the common man” on the other.

Even before the pandemic, there were warnings that growth of science denialism threatens public health [36], and in a similar vein, during the pandemic, distrust in science was repeatedly reported to be a strong predictor of compliance with health guidelines [37,38]. Science skepticism was also correlated with more willingness to spread COVID-19 conspiracies [39] and related misinformation [40].

There is less clarity about the role of trust in the wisdom of the common man in questionable health practices. In the realm of US political science, this construct is sometimes considered a manifestation of anti-intellectualism, in which it represents a substitute for formal knowledge and expertise [41]. In this view, experts are seen as elite, too distanced from average people, exhibiting certain arrogance and patronizing attitudes [42]. Such anti-intellectualism and turning to the plain wisdom of the common man were described as the core of the public’s defiant response to the pandemic [43]. As it is described, however, this is a very (north)American phenomenon, and it has not been cross culturally validated. It is typically contrasted to trust in science, or even measured simply as lack of trust in scientific authorities (as in [43]). The relation between trust in science and trust in folk wisdom could, nevertheless, be more complex. They could be unrelated, but also positively related (even if it seems contradictory), in a so-called polyphasic or “high entropy” representational field [44,45].

A similar, albeit narrower concept of medical folk wisdom has been related to devaluing medical expertise, but not to health behaviors [46], and there is no evidence of predictiveness of trust in common man for pandemic health behavior, neither adhering to recommendations nor resorting to alternative practices.

There is reason to assume that less trust in official authorities would leave people more open to alternative scenarios—conspiratorial beliefs are by definition alternatives to the official ones, and, in the pandemic, the official authorities were clear that relying on natural immunity would not suffice in fighting the virus. These beliefs could, in turn, make it more probable for a person to not adhere to official recommendations, i.e. vaccination, and to resort to other, alternative health practices. As previously discussed, it is less clear what the consequences of more trust in wisdom in common man would be. Drawing from the existing empirical evidence (albeit only from the US and thus maybe not culturally universal), one might assume it could follow a similar path: more trust in the wisdom of the common man could be related to more conspiratorial beliefs and higher endorsement of the appeal to nature bias and thus to more questionable health practices.

We first set out to explore how trust in science and the wisdom of the common man, and the two types of epistemically suspect beliefs–conspiratorial beliefs about COVID-19 and the appeal to nature bias–relate to each other, and how they relate to a. the current COVID-19 vaccination status and b. the use of pseudoscientific health practices as protection against COVID-19. We further tested whether these two types of trust impacted vaccination status/pseudoscientific practices through the two types of epistemically suspect beliefs.

## Study 1

In the first study, we measured only vaccination status as an outcome. We expected the two types of epistemically suspect beliefs–conspiratorial beliefs and the appeal to nature bias–to be positively related (H1). Based on considerations of the two epistemic authorities and their relationship in previous research, we expected that the two types of trust–in science and in the wisdom of the common man–would be negatively related (H2). Finally, we hypothesized that trust in science and trust in the wisdom of the common man would contribute to prediction of vaccination status through conspiratorial beliefs (H3a) and through the appeal to nature bias (H3b).

### Methods

#### Ethics statement

The study was approved by the Institutional review board of the Department of Psychology, Faculty of Philosophy, University of Belgrade, Serbia (protocol number: #2021–001); the participants provided their written consent. The protocol strictly followed the Declaration of Helsinki, and the data was collected and stored abiding by the GDPR regulations (for detailed IPSOS data policy consult: https://www.ipsos.com/en/privacy-data-protection)

#### Sampling and data collection procedure

Data collection was conducted through Ipsos Serbia’s monthly omnibus research, during May 2021, on a total of 1001 respondents, representative of the adult population of Serbia (95% confidence interval for incidence of 50% on the sample size of 1000 is +/- 3.2%). We used a mixed-mode approach—80% of the data was collected via face-to-face computer-assisted interviews, while the other 20% was collected via online interviews through the Ipsos Online Panel. It was a stratified three-staged probability sample, with the target population of citizens of Serbia aged over 18 years (the sampling frame was based on the data from the 2011 population census). It was stratified by the type of settlement, and by six geo-economical regions. The data was weighted by education to better match the population.

Table 1 details the socio-demographic breakdown of the sample, as well as the official population estimates for Serbia.

**Table 1:** Socio-demographic breakdown of the sample—Study 1.

	n	%	2021 Population Estimates^a^
Gender			
Female	522	52.2	51.3
Male	479	47.8	48.7
Age			
18–29	184	18.4	13.2
30–39	165	16.5	13.6
40–49	165	16.5	14.3
50–65	285	28.5	21.7
66+	202	20.2	19.8
Education level			
Primary education or lower	280	27.9	34.4
Secondary (high school) education	547	54.6	48.9
Higher education	175	17.4	16.2
Type of settlement			
Urban	583	58.2	61.4
Rural	418	41.8	38.6

^a^ The percentages shown are population estimates for 2021 according to the Statistical Office of the Republic of Serbia, except the education estimates, which are based on the available 2011 census data. Those percentages do not add up to 100 due to missing data.

### Materials

*Belief in COVID-19 conspiracy theories*. Belief in COVID-19 conspiracy theories was assessed via two items: “*The so-called COVID-19 pandemic is nothing more than a smokescreen for covert actions of powerful forces”* and “*It is clear that the pharmaceutical industry is behind this pandemic”*. The participants assessed the truthfulness of the items on a 9-point Likert type scale, ranging from 1 (*completely false*) to 9 (*completely true*). The score was calculated as a mean of answers on the two items. We had additionally included a negatively reflected item “*I think the public is correctly informed about the origins of the coronavirus*”, however, we excluded it from the final score since it had a poor corrected item-total correlation (*r* = .159). Upon exclusion, the internal consistency of the scale was good α = .75.

*Appeal to nature bias regarding COVID-19*. The appeal to nature bias was assessed with two items: *“COVID-19 can be beaten with natural remedies and nutrition*.*”* and “*The coronavirus is harmless for those with a strong immunity*”. The participants indicated the truthfulness of the items on a scale ranging from 1 (*completely false*) to 9 (*completely true*). The internal consistency of the two items (α = .66), allowed us to average them in the final score.

*Trust in science*. Trust in science was assessed with one item: *“When it comes to dealing with the coronavirus and the knowledge about it*, *how much trust do you have in science and scientific findings*?*”*. The participants answered using a 9-point scale, from 1 (*I do not trust it at all*) to 9 (*I trust it completely*).

*Trust in the wisdom of the common man*. Trust in the common man was assessed with the item *“When it comes to dealing with the coronavirus and the knowledge about it*, *how much trust do you have in the wisdom of the common man*?*”*. Answers were given on a scale ranging from 1 (*I do not trust it at all*) to 9 (*I trust it completely*).

*Vaccination status*. We assessed vaccination status by asking the participants *“Did you get vaccinated against COVID-19 (with at least one dose)*?*”*. Participants answered using a scale from 1 (*No*, *and I will surely not get vaccinated*), 2 (*No*, *and I will probably not get vaccinated*), 3 (*No*, *and I am not sure if I will get vaccinated or not*), 4 (*No*, *but I will probably get vaccinated*), 5 (*No*, *but I will surely get vaccinated*), 6 (*No*, *but I have signed up for vaccination*^1^) to 7 (*Yes*). (^1^ At this point in time, it was required to sign up to get vaccinated in Serbia in order to get a vaccination appointment.) We additionally included a category for those for whom the vaccine is not recommended. This category made up 5.8% of the sample and we coded these answers as system missing. We opted for a 7-point scale rather than a binary one given that the study was done mid-year 2021 when the vaccines were still becoming more widely available. As such, those who had a strong intent to get vaccinated might not have logistically been able to do so yet, and could not thus be lumped together with those who strongly opposed getting vaccinated against COVID-19. It was important to have those differences among the ones not (yet) vaccinated reflected in the measure given the particular context. We label the measure “vaccination status” to more precisely illustrate its nature, given that it is a combination of a behavioral and intentional measure.

### Results

All materials, data and syntax are available on Open Science Framework (OSF) project page: https://osf.io/5fuzn/.

### Endorsement of COVID-19-related epistemically suspect beliefs

We first examined the population prevalence of the epistemically suspect beliefs (Table 2 details the distribution of answers).

**Table 2:** Distribution of answers on the endorsement of epistemically suspect beliefs—Study 1.

	Not True / Does Not Trust (1–4)	Unsure (5)	True / Trusts(6–9)
*Conspiratorial beliefs*			
The so-called COVID-19 pandemic is nothing more than a smokescreen for covert actions of powerful forces.	37%	26%	37%
It is clear that the pharmaceutical industry is behind this pandemic.	32%	27%	41%
*Appeal to nature bias*			
COVID-19 can be beaten with natural remedies and nutrition.	45%	21%	34%
The coronavirus is harmless for those with a strong immunity.	43%	19%	38%
*When it comes to dealing with the coronavirus and knowledge about it*, *how much trust do you have in*.* *.* *.			
… science	12%	14%	74%
… the wisdom of the common man	33%	25%	42%

*Note*. The percentage of participants indicating a certain answer on each of the items—Not true (answers 1 through 4), Unsure (answer 5), True (answers 6 through 9).

Conspiracy theories about the pandemic were endorsed by a significant proportion of the population, with more than a third finding them more likely to be true than not. Similarly, the appeal to nature bias, while slightly less present than conspiratorial beliefs, was also observed in more than a third of the population. Overall, this suggests that, while not prevalent, epistemically suspect beliefs are far from marginalized.

### Trust in science and the wisdom of the common man

Table 2 also details the distribution of answers regarding trust in two different epistemic authorities—science and the wisdom of the common man.

Overall, trust in science was high, with a vast majority of the population indicating that they trust that science is able to deal with the pandemic, and only 12 percent indicating the opposite. Trust in the wisdom of the common man was more evenly distributed: 40% claimed to trust it, whilst 33% did not. Additionally, the pattern of results suggested that the appeal to nature bias was more present in younger people, who also tended to trust science less (for full breakdown consult S1 Appendix).

### Relations between epistemically suspect beliefs, trust and vaccination status

Next, we examined the relations between belief in COVID-19 related conspiracy theories, the appeal to nature bias, trust in science and the wisdom of the common man and vaccination status (Table 3). We transformed all of the variables using the Rankit rank-based normalization for subsequent analyses [47], but we report non-transformed descriptives for ease of interpretation.

**Table 3:** Means, standard deviations and Pearson correlations—Study 1.

	M	SD	1	2	3	4
1. Belief in COVID-19 CTs	5.07	2.58				
2. Appeal to nature bias	4.59	2.6	.45***			
3. Trust in science	7.01	2.35	-.26***	-.25***		
4. Trust in the wisdom of the common man	5.16	2.66	.19***	.25***	.03	
5. Vaccination status	5.26	2.15	-.28***	-.38***	.39***	-.07*

*Note*. N = 1001 for all variables except 5. where N = 943 due to missing values. Correlations significant after Bonferroni-adjusting for all 10 correlations are printed in bold.

* indicates *p* < .05.

*** indicates *p* < .001.

As Table 3 suggests, vaccination status was meaningfully related to all other constructs. As expected, it was most strongly related to the appeal to nature bias and trust in science: negatively to the former and positively to the latter; it was negatively albeit very weakly related to trust in the wisdom of the common man. Additionally, also in line with expectations (H1), belief in COVID-19 conspiracy theories and the appeal to nature bias were moderately positively related, whilst both were correlated with the two types of trust: negatively with science and positively with the wisdom of the common man. Contrary to our expectations (H2), the two types of trust were not related to each other.

*Mediation analyses*. All mediation analyses were run using JASP version 0.16 [48]. We used the maximum likelihood estimator, after performing a listwise deletion of the missing data, given that it was not missing at random. We estimated the confidence intervals using the bootstrap bias-corrected percentile method.

**Fig 1:**
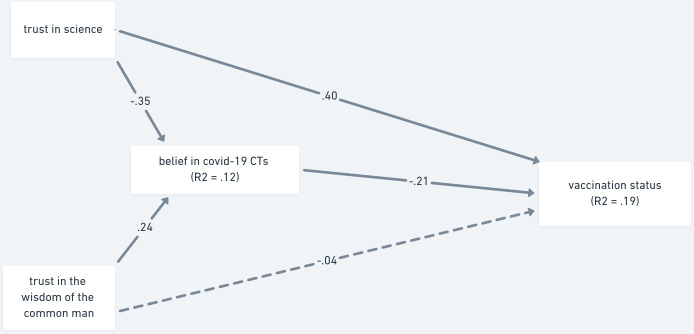
Mediation analysis of trust as a predictor of vaccination status via belief in COVID-19 conspiracy theories—Study 1. *Note*. The paths are shown with standardized estimates. Non-significant paths are shown with dashed lines.

In the first model (Fig 1), we tested whether the two types of trust predict vaccination status through belief in conspiracy theories. Epistemically suspect beliefs only partially mediated the relationship between trust in science and vaccination status, partially in line with our expectations (H3a). The relation of trust in the wisdom of the common man and vaccination status was, as expected (H3b), fully mediated by epistemically suspect beliefs. Trust in science contributed both directly (β = .400, 95% CI [.320, .475]; *p* < .001) and indirectly through belief in COVID-19 conspiracy theories (β = .074, 95% CI [.048, .104]; *p* < .001) to the prediction of vaccination status. On the other hand, trust in the wisdom of the common man had no direct effect on vaccination status (β = -.040, 95% CI [-.108, .024]; *p* = .220), but contributed to its prediction via belief in COVID-19 conspiracy theories (β = -.052, 95% CI [-.077, -.033]; *p* < .001). The model explained a total of 19% of variance in the vaccination status.

**Fig 2:**
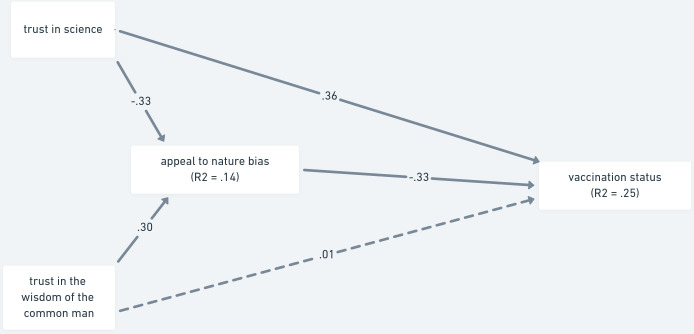
Mediation analysis of trust as a predictor of vaccination status via the appeal to nature bias—Study 1. *Note*. The paths are shown with standardized estimates. Non-significant paths are shown with dashed lines.

When we entered the appeal to nature bias as the mediator in the model (Fig 2), a similar pattern of results emerged. Trust in the wisdom of the common man contributed to the prediction solely through the appeal to nature bias (β = -.099, 95% CI [-.130, -.069]; *p* < .001), while trust in science contributed both directly (β = .363, 95% CI [.284, .441]; *p* < .001) and indirectly (β = .111, 95% CI [.081, .149]; *p* < .001). In total, the model explained 25% of variance in vaccination status.

## Study 2

As our main goal was to explore psychological roots of questionable health practices, we wanted to replicate the findings of the first study, and extend it by including another health outcome in a new sample. To this end, we assessed the frequency of use of different non-evidence-based treatments that were advertised to build protection against the COVID-19. As these types of remedies are often sold as “natural solutions” and/or “traditional cures” [9,49], its use is even more likely to be related to the appeal to nature bias, and further to trust in the wisdom of the common man as an epistemic authority.

We once again expected trust in science and the wisdom of the common man to have an indirect effect on vaccination status through both conspiratorial beliefs (H1a) and the appeal to nature bias (H1b). As for the use of pseudoscientific practices, we hypothesized that both types of trust would contribute to its prediction through conspiratorial beliefs (H2a) and the appeal to nature bias (H2b). Finally, we expected the appeal to nature bias to be a stronger predictor of the use of pseudoscientific practices than the endorsement of conspiratorial beliefs (H3).

### Methods

#### Sampling and data collection procedure

We followed the same sampling and data collection procedure as in Study 1. The sample was representative of the adult population of Serbia with a total of N = 1010 participants; the data was collected in June 2021. Table 4 details the socio-demographic breakdown of the sample, alongside official population estimates for 2021.

**Table 4:** Socio-demographic breakdown of the sample—Study 2.

	n	%	2021 Population Estimates^a^
*Gender*			
Female	534	52.9	51.3
Male	476	47.1	48.7
*Age*			
18–29	179	17.7	13.2
30–39	166	16.4	13.6
40–49	170	16.9	14.3
50–65	278	27.5	21.7
66+	216	21.4	19.8
*Education level*			
Primary education or lower	261	25.8	34.4
Secondary (high school) education	570	56.5	48.9
Higher education	179	17.7	16.2
*Type of settlement*			
Urban	579	57.3	61.4
Rural	431	42.7	38.6

^a^ The percentages shown are population estimates for 2021 according to the Statistical Office of the Republic of Serbia, except the education estimates, which are based on the available 2011 census data. Those percentages do not add up to 100 due to missing data.

#### Materials

*Use of pseudoscientific practices*. The use of pseudoscientific practices was assessed via the frequency of engagement in three types of pseudoscientific practices for protection against the coronavirus: (1) eating garlic, (2) drinking healing concoctions (such as ginger tea, lemon with baking soda, or similar drinks) and (3) eating honey or other bee products. These practices were selected because they were among the most common ones in Serbia based on a previous study [26]. Participants indicated how often they partook in these activities during the pandemic in order to protect themselves from the coronavirus on a scale ranging from 1 (*never*) to 5 (*very often*). We calculated the score by taking the mean of answers on all three items. The scale’s internal consistency was satisfactory α = .65.

We also assessed belief in COVID-19 conspiracy theories (α = .75), the appeal to nature bias regarding COVID-19 (α = .61) and vaccination status using the same measures as in Study 1.

### Results

All materials, data and syntax are available on the OSF project page: https://osf.io/5fuzn/ https://osf.io/5fuzn/?view_only=7ad27e0f55364bc28498ec6a6a234087.

### Endorsement of COVID-19-related epistemically suspect beliefs

As in Study 1, conspiracy beliefs were more present in the population compared to the appeal to nature bias, but both types of beliefs were slightly less endorsed than in Study 1 (Table 5).

**Table 5:** Distribution of answers on the endorsement of epistemically suspect beliefs—Study 2.

	Not True / Does Not Trust (1–4)	Unsure (5)	True / Trusts (6–9)
*Conspiratorial beliefs*			
The so-called COVID-19 pandemic is nothing more than a smokescreen for covert actions of powerful forces.	39%	23%	38%
It is clear that the pharmaceutical industry is behind this pandemic.	35%	22%	43%
*Appeal to nature bias*			
COVID-19 can be beaten with natural remedies and nutrition.	53%	18%	29%
The coronavirus is harmless for those with strong immunities.	48%	18%	34%
*When it comes to dealing with the coronavirus and knowledge about it*, *how much trust do you have in*.* *.* *.			
. . .science	13%	15%	72%
. . .the wisdom of the common man	41%	26%	33%

*Note*. The percentage of participants indicating a certain answer on each of the items—Not true (answers 1 through 4), Unsure (answer 5), True (answers 6 through 9).

### Trust in science and the wisdom of the common man

Table 5 also details the percentage of the population that has (vs. the percentage that does not have) trust in science and the wisdom of the common man.

Compared to Study 1, trust in science remained high, while trust in the wisdom of the common man was slightly less prevalent, with only a third of the population indicating that they trust it when it comes to dealing with the pandemic.

Similar to Study 1, we found that the appeal to nature bias mostly decreases with age, while the opposite is true for trust in science (S1 Appendix).

### Use of pseudoscientific practices as protective measures

While the use of pseudoscientific practices was not widespread in the population, there was still a significant portion of people who resorted to it (Table 6). More than 50 percent of the participants indicated that they at least sometimes ate garlic or bee products to protect themselves from the virus, while the least prevalent practice was the consumption of healing concoctions, (42% of the population used them at least sometimes). However, the majority still claimed they have never resorted to any of the three pseudoscientific practices.

**Table 6:** Distribution of answers on the frequency of use of pseudoscientific practices—Study 2.

	Never	Rarely	Sometimes	Often	Very often
*During the pandemic*, *how often have you done the following in order to protect yourself from coronavirus*?					
Consumed garlic	37%	12%	22%	16%	13%
Drank healing concoctions (such as ginger tea, lemon with baking soda, or similar drinks)	48%	9%	18%	15%	10%
Consumed honey or other bee products	35%	12%	24%	16%	13%

*Note*. The percentage of participants indicating a certain answer on each of the items.

### Relations between epistemically suspect beliefs, trust, pseudoscientific practices and vaccination status

Table 7 shows means, standard deviations and correlations between epistemically suspect beliefs, trust, pseudoscientific practices and vaccination status. We again transformed all of the variables using the Rankit rank-based transformation and reported the non-transformed ones for descriptives.

**Table 7:** Means, standard deviations and Pearson correlations—Study 2.

	M	SD	1	2	3	4	5
1. Belief in COVID-19 CTs	5.09	2.74					
2. Appeal to nature bias	4.21	2.56	.44***				
3. Trust in science	7.00	2.42	-.27***	-.25***			
4. Trust in the wisdom of the common man	4.69	2.81	.32***	.33***	.00		
5. Pseudoscientific practices	2.49	1.11	.09**	.16***	.04	.14***	
6. Vaccination status	5.05	2.35	-.32***	-.38***	.33***	-.13***	-.02

*Note*. N = 1010 for all variables except 6. where N = 937. Correlations significant after Bonferroni-adjusting for all 15 correlations are printed in bold.

** indicates *p* < 01.

*** indicates *p* < .001.

Relations between variables were of similar magnitude as in Study 1. Pseudoscientific practices show relatively weak relations to most variables, with the exception of trust in science and vaccination status. It was most strongly correlated with the appeal to nature bias. The two types of trust were once again not related. Vaccination status showed the strongest correlation with the appeal to nature bias.

*Mediation analyses*. We ran a total of four mediation analyses, two where the outcome variable was vaccination status and two where we used pseudoscientific practice use as the outcome. In all four models, the predictors were trust in science and the wisdom of the common man. For each of the outcome variables, we fit one model with belief in COVID-19 conspiracy theories as a mediator, and one with the appeal to nature bias as a mediator. We used the same analyses properties as in Study 1.

**Fig 3:**
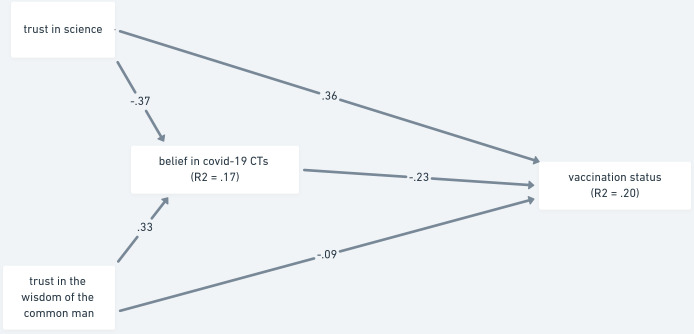
Mediation analysis of trust predicting vaccination status via belief in COVID-19 conspiracy theories—Study 2. *Note*. The paths are shown with standardized estimates. Non-significant paths are shown as dashed lines.

**Fig 4:**
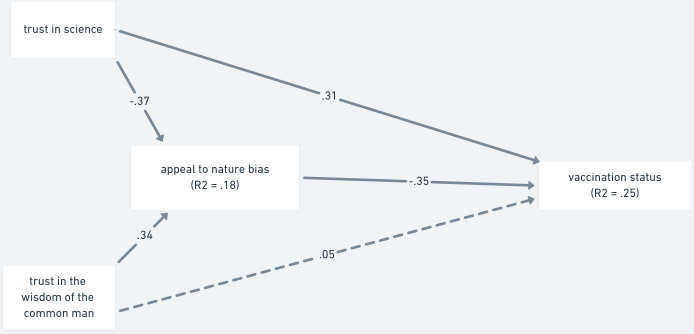
Mediation analysis of trust as a predictor of vaccination status via the appeal to nature bias—Study 2. *Note*. The paths are shown with standardized estimates. Non-significant paths are shown as dashed lines.

*Vaccination status as the outcome*. Firstly, we tested the same models as in Study 1 (Figs 3 and 4). Partially in line with our expectations (H1a), when belief in COVID-19 conspiracy theories was entered as a mediator, trust in science contributed both directly (β = .357, 95% CI [.277, .439]; *p* < .001) and through belief in COVID-19 conspiracy theories (β = .087, 95% CI [.060, .122]; *p* < .001). Similarly, trust in the wisdom of the common man had both a significant direct (β = -.094, 95% CI [-.170, -0.029]; *p* = .008) and indirect effects (β = -.077, 95% CI [-.111, -0.052]; *p* < .001) on vaccination status. The model explained 20 percent of variance of the outcome variable.

Next, we tested the same model, but with the appeal to nature bias as the mediator (Fig 4, H1b), and fully replicated the pattern observed in Study 1. Trust in science contributed both directly (β = .315, 95% CI [.237, .394]; *p* < .001) and indirectly (β = .129, 95% CI [.097, .173]; *p* < .001) to predicting vaccination status. In turn, wisdom of the common man contributed to the prediction through the appeal to nature bias (β = -.120, 95% CI [-.153, -0.090]; *p* < .001), but not directly (β = -.051, 95% CI [-.112, .018]; *p* = .139). In total, the model explained 25% of variance in vaccination status.

*Use of pseudoscientific practices as the outcome*. We proceeded to test the same models, but with use of pseudoscientific practices as the outcome.

**Fig 5:**
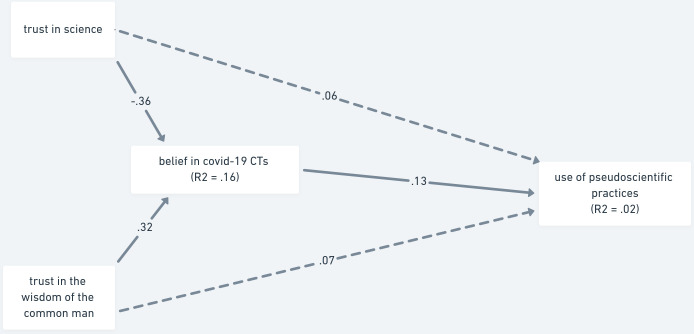
Mediation analysis of trust as a predictor of use of pseudoscientific practices via belief in COVID-19 conspiracy theories—Study 2. *Note*. The paths are shown with standardized estimates. Non-significant paths are shown as dashed lines.

When belief in COVID-19 conspiracy theories was entered as the mediator (Fig 5), the model explained only a total of two percent of the variance of the outcome variable. None of the direct paths was significant, however, as expected (H2a), both trust in science (β = -.045, 95% CI [-.073, -.020]; *p* < .001) and trust in the wisdom of the common man (β = .041, 95% CI [.018, .068]; *p* < .001) had an indirect effect on the use of pseudoscientific practices through belief in COVID-19 conspiracy theories.

**Fig 6:**
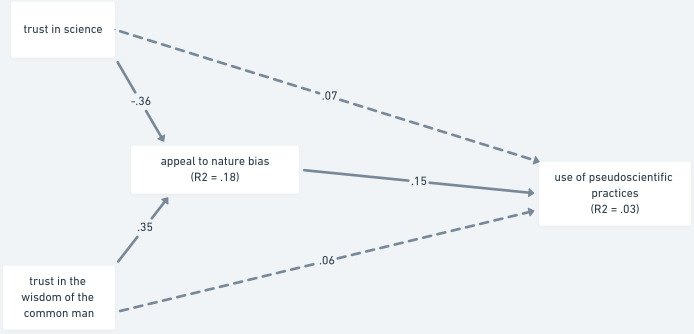
Mediation analysis of trust as a predictor of vaccination status via the appeal to nature bias—Study 2. *Note*. The paths are shown with standardized estimates. Non-significant paths are shown as dashed lines.

Finally, we tested the same model, but with the appeal to nature bias as the mediator (Fig 6). As with the previous model and as we expected (H2b) the relation between trust in science and use of pseudoscientific practices was fully mediated by the appeal to nature bias (β = -.055, 95% CI [-.082, -.027]; *p* < .001), as was the relation between trust in the wisdom of the common man and the use of pseudoscientific practices (β = .053, 95% CI [.024, .080]; *p* < .001), explaining three percent of the variance of the use of pseudoscientific practices.

In line with our prediction (H3), the appeal to nature bias had a slightly stronger correlation to the use of pseudoscientific practices than conspiratorial beliefs. The model with the appeal to nature bias as a mediator explained more variance as well, however this difference was minor.

## Discussion

Across two studies on nationally representative samples, we explored how trust in science and trust in the wisdom of the common man contributed to the prediction of questionable health practices through epistemically suspect beliefs.

We repeatedly observed high trust in the ability of science to deal with the ongoing pandemic, with more than 70 percent of the population indicating they trust science more than they do not, whilst wisdom of the common man was trusted by around one-third. When evaluating this finding, one should take into account that these were self-reported measures, thus potentially sensitive to socially desirable responding—especially relevant for trust in science. However, epistemically suspect beliefs were endorsed by a significant proportion of the population as well: conspiratorial beliefs by 40 percent, and the appeal to nature bias regarding COVID-19 by more than 30 percent. In line with previous research, the two types of beliefs were moderately positively correlated, indicating that they tend to cluster together [17,23]. Our results testify to the role of these beliefs, i.e. to the fact that, although important, trust in science is neither only nor solely a direct precursor of pandemic-related health behaviors.

First, the appeal to nature bias had the strongest correlation to both vaccination status and the use of pseudoscientific practices. The youngest participants who were most prone to appeal to nature were also the ones least vaccinated. Second, trust in science both directly and indirectly predicted the vaccination status, corroborating recent findings [50], whilst trust in the wisdom of the common man did so only indirectly. As for their relation to the use of pseudoscientific practices to protect oneself from COVID-19, it was fully mediated by conspiratorial beliefs and the appeal to nature bias. This might be due to the fact that trust in science is a better-defined construct and has more content overlap with vaccination status. Even though it was widely used in public discourse, the term “wisdom of the common man” is more vague, and can encompass different areas of knowledge. We also found no significant correlation between trust in science and trust in the wisdom of the common man, implying that relying on folk wisdom does not necessarily lead to the devaluation of experts and science and that it cannot be equated to anti-intellectualism. The idea of their potential coexistence has already been raised in the literature [51]: the two types of knowledge were assumed to be drawn upon separately or even simultaneously, depending on the situation.

Our results also revealed a more nuanced relation between the two types of questionable health practices. They were not negatively related as we expected, but unrelated. In addition, while the use of pseudoscientific practices was related to trust in the wisdom of the common man, it was not related to trust in science. Finally, taken together, our models were better at predicting non-adhering to recommended health practices (vaccination) than predicting resorting to non-evidence-based practices. Whether this pattern of results could be better explained by the overall image of the TM/CAM practices or by our specific choice of these practices remains to be tested. Namely, TM/CAM practices as a whole are often described as healthier and with less side effects in comparison to the practices recommended by conventional medicine [52]. To capture enough variance in the population, in this research we deliberately focused on practices low in cost, both in terms of resources and risk, but potentially high in benefit. We did observe their relatively widespread use, with 50 percent of the population reporting that, in order to protect themselves from the virus, they consumed garlic or honey at least sometimes. It could thus be the case that these particular practices were perceived as especially harmless and might have been practiced regardless of whether or not a person is adhering to official recommendations, as an added safeguard against the virus.

## Implications

Although we did observe relatively high reported trust in science across our two samples, that does not necessarily imply that our participants are well equipped to evaluate scientific information, assess the evidence base for certain claims, and disentangle the official versus unofficial sources of information, i.e., that they hold a high level of “epistemic vigilance” [53,54]. During the pandemic, this was an especially difficult task, as certain voices from the scientific community spread conspiracy theories about the virus. Long-term efforts in building scientific and digital literacy could equip laypeople with a skillset to address these issues.

However, a pandemic is an emergency that requires more imminent solutions as well. For example, the epistemic authority of the wisdom of the common man could be better exploited in public communication by tying it to scientific content instead, for example via interventions where “the common man” either endorses science and scientists (“It was always wise to listen to people who know more than you”; “I would always take my car to a mechanic for a repair”), or is asked to be a spokesperson for a scientific consensus, testifying to their adherence to recommended practices.

Similarly, although the appeal to nature bias was repeatedly publicly debunked by health experts, it does not seem to be enough—thus, more complex interventions aiming to dispel the idea of natural or strong immunity as a protective factor against the virus should be devised (for example, direct debunking could be complemented with personal narratives [55]); media reports on young and otherwise healthy people suffering from COVID-19 could serve the same purpose.

## Limitations and future research

Our results point to the role of the appeal to nature bias regarding COVID-19 in predicting important health outcomes in the COVID-19 pandemic. Since exploring the latent structure of this construct was out of the scope of this research, we assessed it via only two items. Future studies should include a broader set of items to understand it better, as well as items that are not specifically tied to COVID-19. For example, it is possible that it encompasses two more specific subdimensions, one which reflects the naturalistic fallacy (i.e., all natural things are good), and the other which reflects its consequences (i.e., beliefs that natural immunity is invincible and/or necessarily better than relying on conventional medicine).

Due to the fact that the study was resource-demanding, we measured other constructs with shorter scales, as well. This led to somewhat lower reliability than the standard. It should be noted, however, that the reliability is still adequate given the number of items used, and that despite this, the results seem to be robust across two large, representative samples.

Although we replicated the findings about the predictiveness of COVID-19 related conspiracy theories for health behavior in the pandemic, the appeal to nature bias was a stronger predictor. It could be due to the specificity of conspiratorial content: if the conspiracies were more health-related or even vaccine-related, their predictiveness and the overall predictiveness of the model could be higher. This would be in line with research pointing to different predictiveness of different types of conspiracy theories [56].

We choose two prominent candidates to represent epistemically suspect beliefs. To illustrate there are more such beliefs forming a mindset, future studies could add more and test their interrelatedness. For example, the role of proneness to type 1 error bias, i.e., minimizing the false positives as they are costlier to make [26], or the role of “doublethink”, i.e., proneness to simultaneously holding incompatible beliefs [45] could be tested. In addition to testing a broader set of this type of beliefs which makes people more vulnerable to avoiding recommended behaviors and resorting to non-evidence-based ones, the design could also incorporate potentially protective constructs that could make people more resistant to these practices. That could be more factual knowledge about the virus or general proneness to analytical thinking.

Future studies should also aim to capture a more diverse range of pseudoscientific practices and include ones more likely to cause side effects or divert from the official treatment, as well as ones more costly in terms of resources. Additionally, focusing on specific populations that might be more prone to extreme pseudoscientific practices could provide better insight as to how these practices relate to each other, to epistemically suspect beliefs and the reliance on conventional medicine in general.

Our results reveal that “wisdom of the common man” is a somewhat vague and culturally sensitive construct. To map it more precisely, and test its invariance, it should be assessed cross-culturally, along with potentially overlapping constructs such as thinking styles, ideological left-right preference, anti-paternalism and individualism/collectivism. It would also be informative to include other epistemic sources, such as religious authorities, and contrast them with the two assessed in our studies.

Finally, the data from these two studies is correlational so it cannot speak of causality. Experimental studies should therefore explore the effects of manipulating trust in epistemic authorities, especially in the wisdom of the common man on a range of health behaviors, and the proposed mediation of the effect by epistemically suspect beliefs.

## Conclusion

This study lends further support to a growing body of work suggesting that conspiratorial beliefs shape pandemic-related health behavior [21], and adds the appeal to nature bias as another epistemically suspect belief that is relevant in this context. Furthermore, it speaks to the importance of epistemic authorities as sources of information: besides once again flagging trust in science, it brings new evidence about the role of trust in the wisdom of the common man. The results reveal the relation between the two is sensitive to the cultural context, and that they need not be mutually exclusive. In addition to recommending long-term efforts aimed at nurturing trust in science and a better understanding of the scientific process, we also offer recommendations for tailoring short-term messaging from public health and media professionals.

## Supporting information

S1 Appendix Supporting information. (DOCX)
