# Supplementary material for: I trust my immunity more than your vaccines: “Appeal to nature” bias strongly predicts questionable health behaviors in the COVID-19 pandemic
Source: PLoS One. 2023 Feb 22;18(2):e0279122. doi: 10.1371/journal.pone.0279122 (PMC9946228; doi:10.1371/journal.pone.0279122)
Supplement: S1 Appendix — (DOCX) [file pone.0279122.s001.docx]

**S1 Appendix**

**S1 Table 1. Distribution of answers on the endorsement of conspiratorial beliefs by age - Study 1.**

|  | Age | | | | |
| --- | --- | --- | --- | --- | --- |
| Conspiratorial beliefs | 18-29 | 30-39 | 40-49 | 50-64 | 65+ |
| *The pandemic is just a smokescreen for covert operations.* |  |  |  |  |  |
| Not True (1-4) | 39 | 41 | 44 | 33 | 32 |
| Unsure (5) | 19 | 22 | 25 | 29 | 31 |
| True (6-9) | 42 | 37 | 31 | 38 | 37 |
|  |  |  |  |  |  |
| *The pharmaceutical industry is behind the pandemic.* |  |  |  |  |  |
| Not True (1-4) | 35 | 39 | 32 | 26 | 30 |
| Unsure (5) | 16 | 27 | 33 | 27 | 31 |
| True (6-9) | 49 | 34 | 35 | 47 | 39 |

*Note.* The percentage of participants indicating a certain answer on each of the items - Not true (answers 1 through 4), Unsure (answer 5), True (answers 6 through 9)

**S1 Table 2. Distribution of answers on the appeal to nature bias by age - Study 1.**

|  | Age | | | | |
| --- | --- | --- | --- | --- | --- |
| Appeal to nature bias | 18-29 | 30-39 | 40-49 | 50-64 | 65+ |
| *COVID-19 can be beaten with natural remedies and nutrition.* |  |  |  |  |  |
| Not True (1-4) | 38 | 47 | 51 | 50 | 40 |
| Unsure (5) | 18 | 21 | 24 | 23 | 20 |
| True (6-9) | 44 | 32 | 25 | 27 | 40 |
|  |  |  |  |  |  |
| *The coronavirus is harmless for those with strong immunities.* |  |  |  |  |  |
| Not True (1-4) | 37 | 43 | 44 | 46 | 43 |
| Unsure (5) | 17 | 18 | 24 | 15 | 22 |
| True (6-9) | 46 | 39 | 32 | 39 | 35 |

*Note.* The percentage of participants indicating a certain answer on each of the items - Not true (answers 1 through 4), Unsure (answer 5), True (answers 6 through 9)

**S1 Table 3. Distribution of answers on trust in science and the wisdom of the common man by age - Study 1.**

|  | Age | | | | |
| --- | --- | --- | --- | --- | --- |
|  | 18-29 | 30-39 | 40-49 | 50-64 | 65+ |
| *Trust in science* |  |  |  |  |  |
| Does Not Trust (1-4) | 17 | 17 | 13 | 10 | 6 |
| Unsure (5) | 19 | 15 | 16 | 13 | 11 |
| Trusts (6-9) | 64 | 68 | 71 | 77 | 83 |
|  |  |  |  |  |  |
| *Trust in the wisdom of the common man* |  |  |  |  |  |
| Does Not Trust (1-4) | 42 | 36 | 30 | 34 | 25 |
| Unsure (5) | 18 | 27 | 27 | 29 | 23 |
| Trusts (6-9) | 40 | 37 | 43 | 37 | 52 |

*Note.* The percentage of participants indicating a certain answer on each of the items - Does Not Trust (answers 1 through 4), Unsure (answer 5), Trusts (answers 6 through 9).

**S1 Table 4. Distribution of answers on the endorsement of conspiratorial beliefs by age - Study 2.**

|  | Age | | | | |
| --- | --- | --- | --- | --- | --- |
| Conspiratorial beliefs | 18-29 | 30-39 | 40-49 | 50-64 | 65+ |
| *Pandemic is just a smokescreen for covert operations.* |  |  |  |  |  |
| Not True (1-4) | 43 | 38 | 46 | 42 | 26 |
| Unsure (5) | 20 | 14 | 18 | 23 | 37 |
| True (6-9) | 37 | 48 | 36 | 35 | 37 |
|  |  |  |  |  |  |
| *The pharmaceutical industry is behind the pandemic.* |  |  |  |  |  |
| Not True (1-4) | 37 | 28 | 40 | 37 | 30 |
| Unsure (5) | 17 | 17 | 20 | 22 | 32 |
| True (6-9) | 46 | 55 | 40 | 41 | 38 |

*Note.* The percentage of participants indicating a certain answer on each of the items - Not true (answers 1 through 4), Unsure (answer 5), True (answers 6 through 9)

**S1 Table 5. Distribution of answers on the appeal to nature bias by age - Study 2.**

|  | Age | | | | |
| --- | --- | --- | --- | --- | --- |
| Appeal to nature bias | 18-29 | 30-39 | 40-49 | 50-64 | 65+ |
| *COVID-19 can be beaten with natural remedies and nutrition.* |  |  |  |  |  |
| Not True (1-4) | 51 | 44 | 60 | 57 | 52 |
| Unsure (5) | 15 | 15 | 17 | 16 | 25 |
| True (6-9) | 34 | 41 | 23 | 27 | 23 |
|  |  |  |  |  |  |
| *The coronavirus is harmless for those with strong immunities.* |  |  |  |  |  |
| Not True (1-4) | 48 | 36 | 53 | 53 | 46 |
| Unsure (5) | 14 | 16 | 17 | 17 | 24 |
| True (6-9) | 38 | 48 | 30 | 30 | 30 |

*Note.* The percentage of participants indicating a certain answer on each of the items - Not true (answers 1 through 4), Unsure (answer 5), True (answers 6 through 9)

**S1 Table 6. Distribution of answers on trust in science and the wisdom of the common man by age - Study 2.**

|  | Age | | | | |
| --- | --- | --- | --- | --- | --- |
|  | 18-29 | 30-39 | 40-49 | 50-64 | 65+ |
| *Trust in science* |  |  |  |  |  |
| Does Not Trust (1-4) | 25 | 16 | 9 | 11 | 5 |
| Unsure (5) | 13 | 19 | 15 | 14 | 13 |
| Trusts (6-9) | 62 | 65 | 76 | 75 | 82 |
|  |  |  |  |  |  |
| *Trust in the wisdom of the common man* |  |  |  |  |  |
| Does Not Trust (1-4) | 51 | 40 | 46 | 37 | 35 |
| Unsure (5) | 20 | 24 | 25 | 27 | 33 |
| Trusts (6-9) | 29 | 36 | 29 | 36 | 32 |

*Note.* The percentage of participants indicating a certain answer on each of the items - Does Not Trust (answers 1 through 4), Unsure (answer 5), Trusts (answers 6 through 9).
